# Small surfactant-like peptides can drive soluble proteins into active aggregates

**DOI:** 10.1186/1475-2859-11-10

**Published:** 2012-01-18

**Authors:** Bihong Zhou, Lei Xing, Wei Wu, Xian-En Zhang, Zhanglin Lin

**Affiliations:** 1 Department of Chemical Engineering, Tsinghua University, One Tsinghua Garden Road, Beijing 100084, China; 2 State Key Laboratory of Virology, Wuhan Institute of Virology, Chinese Academy of Sciences, Wuhan 430071, China

**Keywords:** active protein aggregates, amyloid, peptide-mediated protein aggregation, inclusion bodies, fibrillar structure

## Abstract

**Background:**

Inactive protein inclusion bodies occur commonly in *Escherichia coli *(*E. coli*) cells expressing heterologous proteins. Previously several independent groups have found that active protein aggregates or pseudo inclusion bodies can be induced by a fusion partner such as a cellulose binding domain from *Clostridium cellulovorans *(CBDclos) when expressed in *E. coli*. More recently we further showed that a short amphipathic helical octadecapeptide 18A (EWLKAFYEKVLEKLKELF) and a short beta structure peptide ELK16 (LELELKLKLELELKLK) have a similar property.

**Results:**

In this work, we explored a third type of peptides, surfactant-like peptides, for performing such a "pulling-down" function. One or more of three such peptides (L_6_KD, L_6_K_2_, DKL_6_) were fused to the carboxyl termini of model proteins including *Aspergillus fumigatus *amadoriase II (AMA, all three peptides were used), *Bacillus subtilis *lipase A (LipA, only L_6_KD was used, hereinafter the same), *Bacillus pumilus *xylosidase (XynB), and green fluorescent protein (GFP), and expressed in *E. coli*. All fusions were found to predominantly accumulate in the insoluble fractions, with specific activities ranging from 25% to 92% of the native counterparts. Transmission electron microscopic (TEM) and confocal fluorescence microscopic analyses confirmed the formation of protein aggregates in the cell. Furthermore, binding assays with amyloid-specific dyes (thioflavin T and Cong red) to the AMA-L_6_KD aggregate and the TEM analysis of the aggregate following digestion with protease K suggested that the AMA-L_6_KD aggregate may contain structures reminiscent of amyloids, including a fibril-like structure core.

**Conclusions:**

This study shows that the surfactant-like peptides L_6_KD and it derivatives can act as a pull-down handler for converting soluble proteins into active aggregates, much like 18A and ELK16. These peptide-mediated protein aggregations might have important implications for protein aggregation *in vivo*, and can be explored for production of functional biopolymers with detergent or other interfacial activities.

## Background

Inactive inclusion bodies are commonly formed during the overexpression of heterologous proteins in recombinant hosts such as *E. coli *[1]. Only a limited number of them, often small proteins with no or few cysteine residues, can be recovered through refolding [2]. It has been generally accepted that these nonfunctional inclusion bodies are noncrystalline, amorphous structures [3]. One notable exception was the inclusion bodies of beta-galactosidase obtained from overexpression in *E. coli*, which were found to be biologically active [4]. In recent years, however, several groups have strikingly observed the spontaneous formation of pseudo inclusion bodies which are active, when the target proteins are fused to an aggregation-prone domain or peptide [5-7]. For example, D-amino acid oxidase from *Trigonopsis variabilis *(TvDAO) fused with a cellulose binding domain from *Clostridium cellulovorans *(CBD_clos_) yielded an enzyme aggregate retaining high specific activity [5]. Similarly, MalE31, an aggregation-prone variant of the maltose-binding protein, and a β-amyloid peptide variant Aβ(F19D) have also been used as fusion partners for inducing active protein aggregates [6,7]. These domains and peptide presumbly provide the specific self-associating modules for the fusion protiens and thus drive the target proteins into aggregates [5].

In our previous studies [8,9], we unexpectedly found that a short amphipathic α helical octadecapeptide 18A (EWLKAFYEKVLEKLKELF) was able to induce several normally soluble proteins into active protein aggregates when expressed in *E. coli*. The fourier transform infrared (FTIR) spectra of the 18A peptide induced protein aggregates revealed enhanced α helical secondary structures, suggesting it was the assoication of the 18A peptide that led to the formation of pseudo inclusion bodies. Since amphipathic alpha peptides are abundent in protein strucutres [10], this observation might have implications for protein aggregation in general for biological systems. We subsequently considered whether there were peptides of a different structure other than alpha that could act as a pulling down handler, and a second peptide, ELK16 (LELELKLKLELELKLK) which is beta sturcure in nature was found to have a similar property [9].

To study the generality of this short-peptide induced protein aggregation, in this work we set out to test a third type of small peptides, i.e., surfactant-like peptides that were designed to mimic surfactants and normally do not resemble either α-helical or β-sheet structure [11,12]. These peptides consist of a hydrophobic tail and a hydrophilic head similar to surfactant molecules, and can spontaneously form nanostructures in aqueous solution. To this end, we attached three of such surfactant-like peptides (L_6_KD, L_6_K_2_, DKL_6_) to the carboxyl termini of model proteins, and expressed the fusions in *E. coli*. Most of the fusion proteins were indeed found to be largely insoluble but retained high biological activity, showing that terminally attached surfactant-like peptides can also drive proteins into biologically active pseudo inclusion boides in *E. coil*.

## Results

### Surfactant-like peptides induced proteins to form active protein aggregates

**Figure 1:**
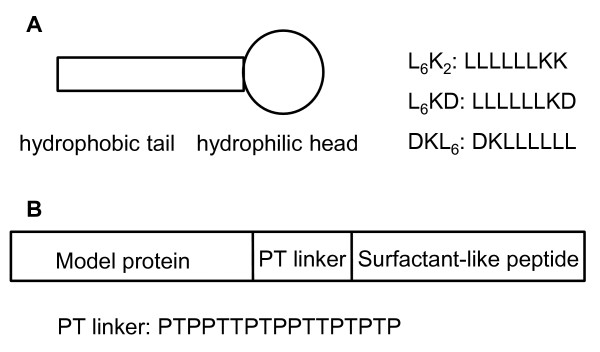
**Schematics for surfactant-like peptide and fusion protein constructs**. (A) The sequences of the surfactant-like peptides used in this study. (B) Genetic constructs of the surfactant-like peptide fusion proteins. Four model proteins: *Aspergillus fumigatus *amadoriase II (AMA), *Bacillus subtilis *lipase A (LipA), *Bacillus pumilus *xylosidase (XynB), and green fluorescent protein (GFP); linker, PTPPTTPTPPTTPTPTP.

The surfactant-like peptides normally contain a hydrophobic tail and a hydrophilic head. We selected or re-designed three short peptides L_6_K_2_, L_6_KD, and DKL_6 _based on the literature (Figure 1) [11,12]. The hydrophobic moiety of all three peptides is six leucines. Leucine has a large hydrophobic side chain, and can pack with each other more easily than other hydrophobic peptides [11]. The hydrophilic moiety of the molecule is two charged amino acids, the positively charged lysine and/or the negatively charged aspartic acid [11], with a total length of 2-3 nm in the extended conformation [11]. Starting from L_6_K_2_, to assess the charge effect, we replaced one of the lysines with one aspartic acid to yield L_6_KD to neutralize the charge, and to assess the positional effect of the hydrophilic head in relation to the model protein, we reversed the sequence of L_6_KD to yield DKL_6_. L_6_D_2 _was not tested as it was reported that L_6_K_2 _and L_6_D_2 _showed little difference in terms of self-assembly property [11,12].

**Figure 2:**
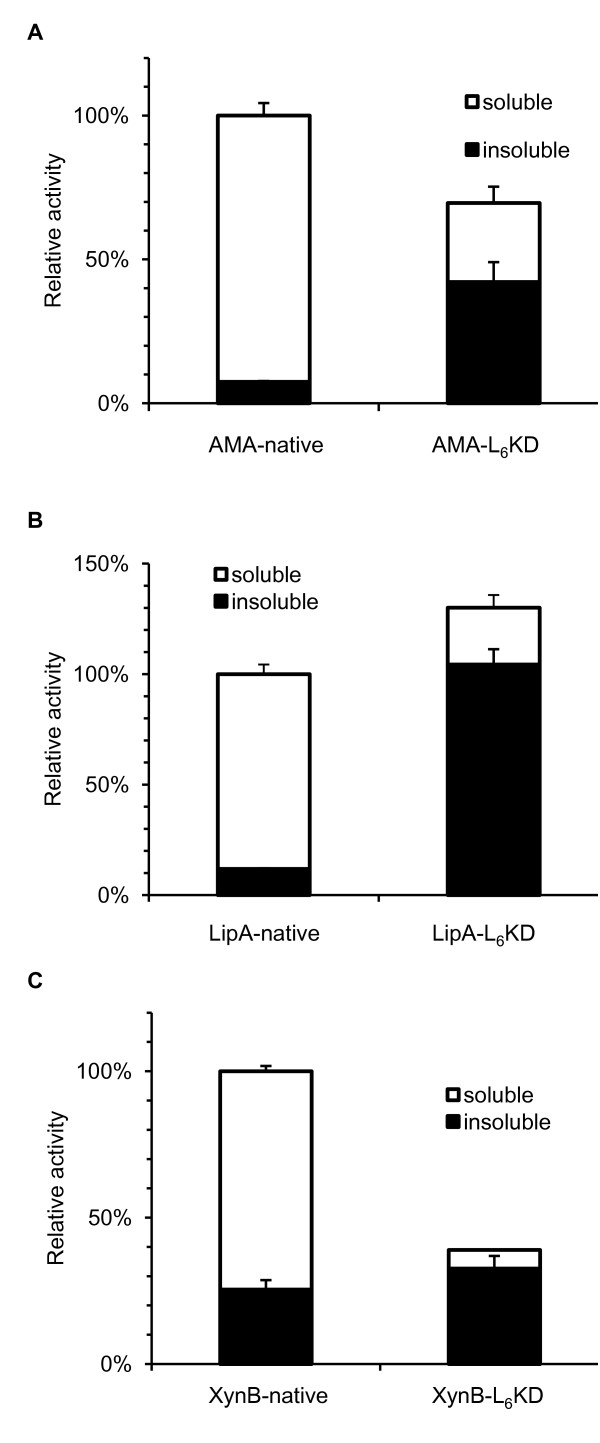
**Distributions of enzyme activities in the soluble and insoluble fractions of cell lysates**. (B) AMA-native and AMA-L_6_KD. (B) LipA-native and LipA-L_6_KD. (C) XynB-native and XynB-L_6_KD. The activities were determined using three independent experiments and normalized to the total activities of the respective native enzyme extracted from a same amount of cells (OD_600_). Standard deviations are also shown.

These three peptides were first fused to the C-terminus of the model protein *Aspergillus fumigatus *amadoriase II (AMA) via a PT linker PTPPTTPTPPTTPTPTP (Figure 1), and expressed in *E. coli *at 30°C. The expression of these fusion proteins had little effect on the cell growth as judged by OD_600 _measurements. Cells were harvested and lysed, and lysates were separated into soluble and insoluble fractions by centrifugation, and analyzed by SDS-PAGE analyses. Most of the fusion proteins were found in the insoluble fractions, in the range of 64%~77% in terms of mass, markedly different from the native protein (~6%). All three fusion proteins in the insoluble fractions were found to be active. For L_6_K_2 _fusion, the activity of the insoluble fraction accounted for about 36.8% of the total activity, and for L_6_KD and DKL_6 _fusions, it was 60.5% (Figure 2A) and 56.1%, respectively. The differences between the mass-based percentages and the activity-based percentages resulted from the different specific activities of the fusion proteins in the soluble and insoluble fractions.

Since AMA-L_6_KD fusion protein produced the highest percentage of active aggregate, this suggested that this peptide was more efficient as a "pulling-down" handler, and thus was chosen for further testing using two additional model proteins, *Bacillus subtilis *lipase A (LipA), and *Bacillus pumilus *xylosidase (XynB) (Figure 2B and 2C). In agreement with the above results, both the LipA-L_6_KD and XynB-L_6_KD fusion proteins formed obvious active aggregates. For LipA-L_6_KD, the insoluble fraction accounted for about 80.2% of the total activity, and for XynB-L_6_KD, it was 83.7%. The relative specific activities of the insoluble fractions for three fusions were showed in Table 1. For AMA-L_6_KD, it was 92.6% of the native counterpart, and for LipA-L_6_KD and XynB-L_6_KD, it was 30.2% and 25.6%, respectively.

**Table 1:** Enzymatic activities of the fusion proteins produced in *E.coli*

Enzyme	Activity (U/ml)^1^		Percent of activity in insoluble fraction (PDE)^2^	Specific activity (U/mg enzyme)^3^	Specific activity relative to native enzyme (SArN)
				
	Soluble fractions	Insoluble fractions			
AMA-native^4^	734.4 ± 37.5	9.63 ± 2.29	1.3%	1563	100%
AMA-L_6_KD	236.0 ± 49.0	361.7 ± 59.5	60.5%	1447	92.6%
LipA-native^5^	20.1 ± 0. 5	2.7 ± 0.3	12.0%	96	100%
LipA-L_6_KD	5.9 ± 0.6	23.8 ± 3.1	80.2%	29	30.2%
XynB-native^5^	398.8 ± 9.7	136.2 ± 17.0	25.4%	1286	100%
XynB-L_6_KD	34.0 ± 0.8	174.5 ± 22.9	83.7%	329	25.6%

^1^Cells were collected 6 h after IPTG induction. 1 ml soluble enzyme was extracted from 10 OD_600 _of cells; the insoluble fraction was also from 10 OD_600 _of cells and then re-suspended in 1 ml of lysis buffer. Enzymes amounts were calculated based on SDS-PAGE with serial concentrations of BSA as standards. AMA, *Aspergillus fumigatus *amadoriase II; LipA, *Bacillus subtilis *lipase A; XynB, *Bacillus pumilus *β-xylosidase.

^2^Percentage of the activity found in the insoluble fraction relative to the total activity in the cell lysate (soluble and insoluble fractions combined), also referred to as pulling down efficiency (PDE).

^3^For the L_6_KD fusion, the value concerns the enzyme in the insoluble fraction (more specifically, enzyme aggregate); for the native enzyme, the value concerns the enzyme in the soluble fraction.

^4^Data cited form reference [9].

^5^Data cited form reference [8].

### Microscopic analyses of active protein aggregates

**Figure 3:**
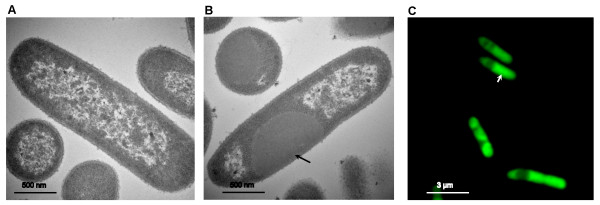
**Intracellular localization of fusion proteins in *E. coli***. (A) and (B), TEM microscopic images for AMA-native and AMA-L_6_KD, respectively. (C) Confocal fluorescent micrograph for GFP-L_6_KD. Size bars are also shown.

To study the intracellular locations of these pseudo inclusion bodies, transmission electron microscopic (TEM) analyses were performed for recombinant cells expressing the fusion protein AMA-L_6_KD. From the TEM images (Figure 3A and 3B), cytoplasmic inclusion bodies were clearly observed for AMA-L_6_KD, with the diameters of about several micrometers. Further studies showed that the fusion proteins LipA-L_6_KD and XynB-L_6_KD had a similar pattern of aggregation (data not shown).

To further confirm the cellular locations of the active protein aggregates, we similarly constructed GFP-L_6_KD fusion, and subjected the *E. coli *cells expressing GFP-L_6_KD to confocal fluorescence microscopic analyses. As shown in Figure 3C, clearly a localized pattern of fluorescence distribution was observed in the recombinant cells, different from the cells expressing native GFP in which a uniformed distribution of fluorescence was observed [9].

### Analyses of amyloid-like properties

**Figure 4:**
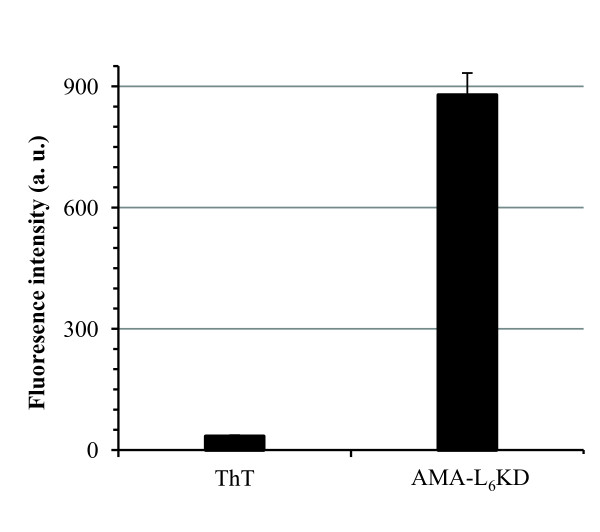
**Binding of AMA-L_6_KD aggregate to thioflavin T (ThT)**. The histograms show the fluorescence intensity of ThT at 480 nm (excited at 445 nm) in the presence and in the absence of AMA-L_6_KD aggregate, in arbitrary units (a. u.).

**Figure 5:**
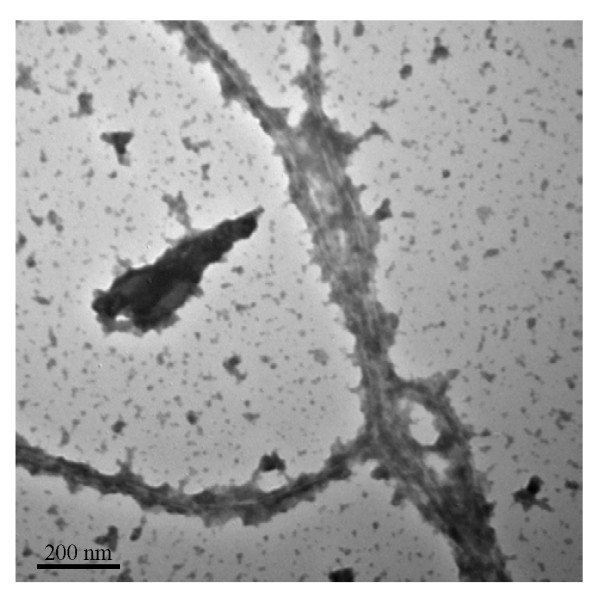
**Fibrillar structure of AMA-L_6_KD protein aggregate**. The micrograph shows the fibers of AMA-L_6_KD aggregate after proteolytic treatment by protease K. Size bar is also shown.

Recently, several research groups reported that inclusion bodies formed in *E. coli *may contain amyloidal structures [13-16] commonly found in protein deposits associated with diseases such as Alzheimer's disease [17-19]. Amyloids characteristically contain fibril-like cores and bind to specific dyes such as thioflavin T (ThT) and Congo red (CR) [18,20,21]. To gain an insight into the molecular mechanism in the surfactant-like peptide-mediated protein aggregation observed in our study, we thus explored the structural property of AMA-L_6_KD aggregate by using thioflavin T and Congo red. Thioflavin T will exhibit a significantly enhanced fluorescence at 480 nm relative to free dye upon binding to amyloid fibrils [21]. As shown in Figure 4, the ThT binding assay for AMA-L_6_KD clearly resulted in a 24-fold increase in the fluorescence at 480 nm (Figure 4). The CR binding assay also showed a shift of absorbance maximum to about 508 nm, and a band at about 541 nm in the differential spectrum, characteristic of binding of Congo red to amyloids (data not shown) [20]. We further used TEM coupled with protease K digestion to explore the morphology of the AMA-L_6_KD aggregate [14,15]. As shown in Figure 5, irregularly organized fibrils can be clearly observed, suggesting an amyloid-like structure for the aggregate.

## Discussion

This study demonstrates that terminally attached surfactant-like peptides like L_6_KD can convert soluble proteins into active aggregates, with specific activities relative to the native counterparts ranging from 25% to 92%. As aggregation inducers, L_6_K_2_, L_6_KD, DKL_6 _are only eight residues in length, even shorter than the peptides 18A (18 residues in length) or ELK16 (16 residues in length). Judging from the activities of the insoluble fractions for their fusions with AMA as the model protein, it seems that a hydrophilic head with a neutral charge (as in the case of AMA-L_6_KD or AMA-DKL_6_) is better at promoting active protein aggregation than a hydrophilic head with a charge (as in the case of AMA-L_6_K_2_). Notably however, the position of the hydrophilic head in relation to the target protein in the fusion, i.e., between the target protein and the hydrophobic tail (as in the case of AMA-DKL_6_) or toward the C-terminal end of the hydrophobic tail (as in the case of AMA-L_6_KD), makes much less of a difference.

Judging from the microscopic analyses, the morphology of these fusion aggregates is similar to that of those induced by aggregation-prone domains and peptides as reported earlier [7-9]. While the exact structural detail by which these surfactant-like peptides drive the target proteins into active aggregates remain to be determined, our analysis of the AMA-L_6_KD aggregate following digestion of protease K revealed fibril-like structures (Figure 4), and the positive binding assay results with amyloid-specific dyes suggest that the AMA-L_6_KD aggregate possesses amyloid-like structures. This is consistent with the view that these pseudo inclusion bodies may contain amyloid-like structures [13-15,17].

Recent studies have shown that, in addition to aggregation-prone domain such as the cellulose binding domain CBD_clos _[5], several peptides can also induce the formation of protein aggregates or inclusion bodies *in vivo*, such as the human β-amyloid peptide Aβ42(F19D) [7], a modified apolipoprotein A-I mimetic amphipathic peptide 18A [8], and the ELK16 peptide derived from EAK16 [9]. These peptides are alpha or beta structure in nature. Our surfactant-like peptides provide a third type of peptide structure beyond alpha or beta that can drive proteins into active aggregates. This peptide-mediated protein aggregation might have important implications for protein aggregation *in vivo*, and for protein aggregation-related diseases. The active protein aggregation induced by these peptides has potential biotechnological applications. For example, it can be used to design a facile expression and purification scheme for protein [22,23], or to obtain active protein aggregates for direct use as biocatalysts [24,25].

Along this line, it is interesting to compare the efficiency of these three short peptides (18A, ELK16 and L_6_KD) as aggregation tags, in terms of both pulling down efficiency (PDE) and specific enzyme activity relative to native enzyme (SArN). As shown in Table 2, ELK16 is a better tag for model enzymes AMA and XynB (LipA lost activity when attached to ELK16), compared with 18A and L_6_KD. L_6_KD is generally comparable with 18A both in terms of PDE and SArN, except that the SArN for LipA-18A fusion aggregate (84%) is much higher than that for LipA-L_6_KD (30.2%). It thus seems that L_6_KD has no superiority in terms of PDE and SArN compared with ELK16 or 18A, except for its smallest length which in some cases may confer advantage for fusion construction albeit in the cost of pulling down efficiency. Additionally, given its unique surfactant property, it is worthwhile to explore the possible use of this tag for design and mass-production of functional biopolymers with detergent or other interfacial activities via microorganism.

**Table 2:** Comparison of three different peptides as aggregation tags

Tag	PDE			SArN		
	
	AMA	LipA	XynB	AMA	LipA	XynB
18A^1^	60.6%	81%	91.3%	88%	84%	21%
ELK16^2^	87.5%	-	94.4%	120%	-	77%
L_6_KD^3^	60.5%	80.2%	83.7%	92.6%	30.2%	25.6%

^1^Data cited from reference [8].

^2^Data cited from reference [9].

^3^Also see Table 1.

## Conclusions

Our study reveals that the presence of surfactant-like peptides can convert fusion proteins into active aggregates *in vivo *which may contain amyloid-like structures. These peptide-mediated protein aggregates may be useful for protein purification, biocatalysis and biosurfactant design and production. Further exploration of this type of protein aggregates may provide new insights into protein aggregation and perhaps related cellular processes and diseases.

## Materials and methods

### Plasmid construction

To construct plasmid pET30a-AMA-L_6_KD, two primers (AMA-up: 5'-TTCTGGACATATGGCGGTAACCAAGTCATC-3', AMA-L_6_KD-down: 5'-ATGAACTCGAGTCAATCTTTCAGCAGCAGCAGCAGCAGCGGCGTCGGGGTTGGGGTG-3', the restriction sites *Nde*I and *Xho*I were underlined, respectively) were used to amplify the gene encoding AMA-L_6_KD from the previously constructed plasmid pET30a-AMA-C18 [8]. Then the amplified DNA fragment was digested with *Nde*I and *Xho*I, and inserted into similarly digested plasmid pET30a (+) (Novagen) to yield pET30a-AMA-L_6_KD. Plasmids pET30a-AMA-L_6_K_2 _and pET30a-AMA-DKL_6 _were similarly constructed using primers AMA-L_6_K_2_-down (5'-ATGAACTCGAGTCATTTTTTCAGCAGCAGCAGCAGCAGCGGCGTCGGGGTTGGGGT-3') and AMA-DKL_6_-down (5'-ATGAACTCGAGTCACAGCAGCAGCAGCAGCAGTTTATCCGGCGTCGGGGTTGGGGTG-3'). Plasmids pET30a-Lip A-L_6_KD, pET30a-XynB-L_6_KD and pET30a-GFP-L_6_KD were obtained by replacing the sequence encoding AMA in pET30a-AMA-L_6_KD with that of Lip A, XynB and GFP, respectively.

### Expression and extraction of protein aggregates

*E. coli *BL21 (DE3) (Novagen) was used for all the experiments. The recombinant strains harboring the plasmids were cultured in Luria-Bertani (LB) medium supplemented with 50 mg/l kanamycin at 37°C. Isopropyl β-D-1-thiogalactopyranoside (IPTG, at a final concentration of 0.2 mM) was added to the culture medium at 30°C to induce fusion protein expression, when the cell optical density at 600 nm (OD_600_) reached 0.4-0.6. After 6 h, cells were harvested by centrifugation, and cell pellets were resuspended in lysis buffer (50 mM Tris-HCl, 50 mM NaCl, 5% glycerol, pH 7.2) with a final concentration of 10 OD_600 _per ml. The resuspended cells were lysed by ultrasonication on ice, and the lysates separated by centrifugation. The insoluble fractions were washed twice with 1 ml of lysis buffer, and resuspended again in a same volume of lysis buffer. The amounts of target proteins in all samples were determined densitometrically by denaturing polyacrylamide gel electrophoresis (SDS PAGE, 12%) using bovine serum albumin (BSA) as standard, followed by staining with Coomassie Brilliant Blue G-250. The values of target protein amounts were calculated with Quantity One software (Bio-Rad Laboratories, Hercules, CA).

### Determination of enzyme activities

The enzyme activities in both the soluble and insoluble fractions were assayed with a SPECTRAMAX M2 microplate reader (Molecular Device, CA). The amadoriase activity [26] was measured by monitoring the formation of a quinone dye in a peroxidase-coupling reaction at 555 nm (e = 39.2 cm^2^/mmol) at 37°C. 5 ml of enzyme was added to 175 ml of reaction mixture (100 mM potassium phosphate buffer (pH 8.0), 2.7 purpurogallin units of peroxidase, 0.45 mM 4-aminoantipyrine, 0.5 mM N-ethyl-N-(2-hydroxy-3-sulfopropyl)-m-toluidine (TOOS), and 5.0 mM D-fructosyl-glycine). One unit of amadoriase was defined as the amount of enzyme that produced 1 nmol H_2_O_2 _per min. LipA activity [27] and XynB activity [28] were measured by monitoring the formation of *p*-nitrophenol at 405 nm (e = 18.7 cm^2^/mmol) at 37°C. For lipase, 5 ml of diluted enzyme was added to 175 ml of reaction mixture (50 mM sodium phosphate buffer, pH 8.0; 0.4 mM *p*-nitrophenyl palmitate; 0.2% sodium deoxycholate, and 0.1% gum arabic). For β-xylosidase, 5 ml of enzyme was added to 175 ml of reaction mixture (50 mM phosphate buffer, pH 6.0, 2.5 mM *p*-nitrophenyl β-D-xylopyranoside). One unit of lipase was defined as the amount of enzyme producing 1 μmol of *p*-nitrophenol (pNP) per min while one unit of xylosidase was defined as the amount of enzyme producing 1 nmol of pNP per min.

### Laser scanning confocal microscopic (LSCM) analyses

The cells expressing fusion protein GFP-L_6_KD were cultivated at 23°C for 22 h after induction with 0.2 mM IPTG. Cells were harvested and washed twice with phosphate buffered saline (PBS). The cell pellets were then fixed with 4% paraformaldehyde and photographed at 488 nm using a Zeiss LSM 710 confocal microscope (Carl Zeiss, Germany).

### Transmission electron microscopic (TEM) analyses

TEM was used to analyze the intracellular location and the morphology of protein aggregates. For the intracellular analyses, recombinant cells were harvested after expression 6 h at 30°C and fixed with fixing solution (2.5% glutaraldehyde and 2% osmium tetraoxide, dehydrated). The fixed cells were dehydrated through a graded-ethanol serial dehydration step, and embedded in epoxy resins. The embedded cells were then sectioned into ultrathin slices, stained by stain solution (containing uranyl acetate solution and lead citrate), and observed with a Hitachi H-7650B (Hitachi, Japan) transmission electron microscope at an accelerating voltage of 80 kV. For the morphology analyses [14], extracted protein aggregates were digested with DNAse and RNAse A (25 μg/ml) for 1 h at 37°C in PBS in the presence of 10 mM MgSO_4_. After that, the protein aggregates were washed with 0.5% Triton-X solution and then PBS. The protein aggregates (50 μg/ml) were then digested using protease K (20 μg/ml) at 37°C for 30 min in PBS, and then washed with PBS. The insoluble fractions were resuspended in a same volume of deionized distilled water, and spotted on copper grids for 5 min. The grids were washed with water, and stained with 1% (w/v) aqueous uranyl formate solution. The prepared samples were then analyzed with a Hitachi H-7650B transmission electron microscope at an accelerating voltage of 75 kV.

### Binding assays of amyloid-specific dyes to AMA-L_6_KD

Thioflavin T (ThT) fluorescence assays were measured with a SPECTRAMAX M2 microplate reader (Molecular Device, CA) with an excitation wavelength of 445 nm and an emission range from 475 nm to 570 nm at 37°C [21,29]. The protein aggregate (in a final concentration of 10 μM) was mixed with 10 μM ThT in PBS in a 96-well black plate. For the Congo red (CR) staining experiment [16,30], 10 μM CR in PBS was incubated in the presence or absence of the protein aggregate (in a final concentration of 10 μM) for 20 min at room temperature and the absorbance spectrum form 360 nm to 700 nm was recorded with a Beckman UV/Vis spectrophotometer.

## Competing interests

The authors declare that they have no competing interests.

## Authors' contributions

BZ designed part of the experiments, performed most of the experiments, and prepared the manuscript draft. LX and WW participated in the enzymatic assays and instrumental analyses. ZL and XZ conceived the study, designed and supervised the experiments, and revised the manuscript. All authors read and approved the final manuscript.
